# Exploring Associations of Different Types of Childhood Trauma With Symptomatology in Irritable Bowel Syndrome

**DOI:** 10.1111/nmo.70148

**Published:** 2025-08-24

**Authors:** Tetyana Bureychak, Jenny Sjödahl, Nawroz Barazanji, Gustav Orell, Olivia Book, Rozalyn Simon, Olga Bednarska, Adriane Icenhour, Susanna Walter

**Affiliations:** ^1^ Division of Diagnostics and Specialist Medicine, Department of Health, Medicine and Caring Sciences Linköping University Linköping Sweden; ^2^ Unit of Physiotherapy, Division of Prevention, Rehabilitation and Community Medicine, Department of Health, Medicine and Caring Sciences Linköping University Linköping Sweden; ^3^ Department of Gastroenterology Linköping University Hospital, County Council of Östergötland Linköping Sweden; ^4^ Center for Medical Image Science and Visualization (CMIV) University Hospital Linköping, County Council of Östergötland Linköping Sweden; ^5^ Department of Affective Neuroscience Ruhr University Bochum Bochum Germany

**Keywords:** adverse childhood experiences, childhood trauma, emotional abuse, exposome, irritable bowel syndrome (IBS)

## Abstract

**Background:**

The connection between childhood trauma and irritable bowel syndrome (IBS) is well documented. However, knowledge regarding distinct associations between different subtypes of childhood trauma and both intestinal and extraintestinal symptoms is widely lacking. The current cross‐sectional study aimed to elucidate the impact of different types of childhood trauma on IBS symptomatology.

**Methods:**

In 169 women with moderate to severe IBS and 39 healthy women (HCs), childhood trauma was assessed using the Childhood Trauma Questionnaire. Gastrointestinal, extraintestinal, and psychological symptoms were evaluated via symptom diaries and questionnaires.

**Key Results:**

The overall prevalence of childhood trauma was significantly higher in women with IBS compared to HCs, with odds ratio (OR) 3.41 (95% CI, 1.35–8.60, *p* = 0.009) and with the highest rates observed for emotional and sexual abuse. Overall childhood trauma was positively associated with symptom severity (*r* = 0.305, *p* = 0.016). Among trauma types, emotional abuse was the strongest predictor of IBS, with a 6‐fold increased odds of disorder, OR 6.69 (95% CI, 1.97–22.68, *p* = 0.002). Women with IBS and a history of emotional abuse reported longer episodes of abdominal pain, more defecation urgency, and higher levels of anxiety and depression.

**Conclusions:**

Our findings support the significant role of childhood trauma in IBS pathology and increased symptom burden. They further indicate the specific relevance of emotional abuse influencing not only gastrointestinal symptoms but also extraintestinal and psychological complaints. These findings may help contribute to the identification of a distinct phenotype of IBS patients with a history of traumatic emotional abuse during childhood.

## Graphical abstract

The current cross‐sectional study aimed to elucidate the impact of different types of childhood trauma on IBS symptomatology. Our findings strongly support the significant role of childhood trauma in IBS pathology and indicate the specific relevance of emotional abuse influencing not only gastrointestinal symptoms but also extraintestinal and psychological complaints.

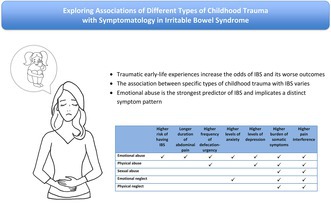

**Summary**

- Childhood trauma is common and plays a significant role in IBS pathology.
- Different types of childhood trauma may have distinct impacts on IBS symptomatology.
- Persons that experienced emotional abuse have higher odds of IBS and share a distinct IBS‐symptom pattern.

## 1 Introduction

Irritable bowel syndrome (IBS) is a common disorder of gut–brain interaction (DGBI) with a global prevalence of approximately 5%–10% and a higher prevalence in women [1, 2]. It is characterized by chronic, recurrent abdominal pain associated with disturbed bowel habits in the absence of any detectable organic disease. The pathophysiology of IBS is complex and not fully understood. Based on an extensive number of studies, a comprehensive disease model of brain–gut–microbiome interaction has emerged [3]. According to it, stress and adverse early life events such as childhood trauma are discussed as part of a wider exposome that may influence brain development and increase central sensitization.

Some of the concepts that have been used to define adverse early life events within the body of IBS research include adverse childhood experiences (ACEs) [4, 5, 6], early adverse life events (EALs) [7, 8, 9, 10], and childhood trauma [11]. Although the concepts overlap partially, the methodological differences between them may affect prevalence numbers, research validity, and comparability of results. Most commonly, adverse childhood experiences refer to those before 18 years of age. Adverse childhood experiences include physical, emotional, and sexual abuse, emotional and physical neglect, and different kinds of household challenges [6]. Early adverse life events are defined as general trauma, physical, emotional, and sexual abuse, household dysfunction, and discordant relationships with the primary caretaker during childhood [7, 10]. Childhood trauma has mostly been studied as child maltreatment, which includes actions of commission (emotional, physical, and sexual abuse) and actions of omission (emotional and physical neglect) [8, 12, 13]. Assessing childhood trauma with the Childhood Trauma Questionnaire (CTQ) allows a more focused evaluation and grading of these five types of child maltreatment, in contrast to measuring exposure to a wider range of adversities.

Childhood maltreatment is widespread and underreported [14]. World Health Organization (WHO) reports that 36% of adults worldwide have experienced emotional abuse as children, 23% were physically abused, 16% were neglected, and 18% of women were sexually abused as children [15]. Girls are, to a higher degree, exposed to all forms of violence compared to boys. In Sweden, results from a recent nationwide study indicate that 43% of schoolgirls have experienced sexual violence, 20% have been exposed to physical abuse, and 18% report emotional abuse [16].

ACEs are associated with increased vulnerability toward developing different kinds of disorders of gut–brain interaction, DGBIs (formerly known as functional gastrointestinal disorders, FGIDs), including IBS [7]. A higher prevalence of childhood trauma has been reported in patients with IBS compared to healthy individuals [4, 5, 7, 8, 17, 18]. According to Drossman et al., patients with FGIDs have experienced more severe forms of abuse, including sexual abuse and life‐threatening physical abuse, when compared to healthy controls [19]. A greater number and increased severity of traumatic childhood experiences are associated with an increased risk of IBS [9], and higher symptom severity [18].

Although childhood trauma has been extensively reported in women with IBS, differences in IBS profiles and symptoms associated with specific kinds of childhood trauma remain poorly understood [8, 20]. By comparing women with IBS with and without various types of childhood trauma, we aimed to explore the associations between specific trauma types and IBS, as well as to elucidate differences in IBS profiles and extraintestinal symptoms. Additionally, we analyzed the overall prevalence of childhood trauma and its subtypes in women with IBS and HCs, and evaluated the association between IBS and childhood trauma.

## 2 Materials and Methods

### 2.1 Study Design and Population

**FIGURE 1:**
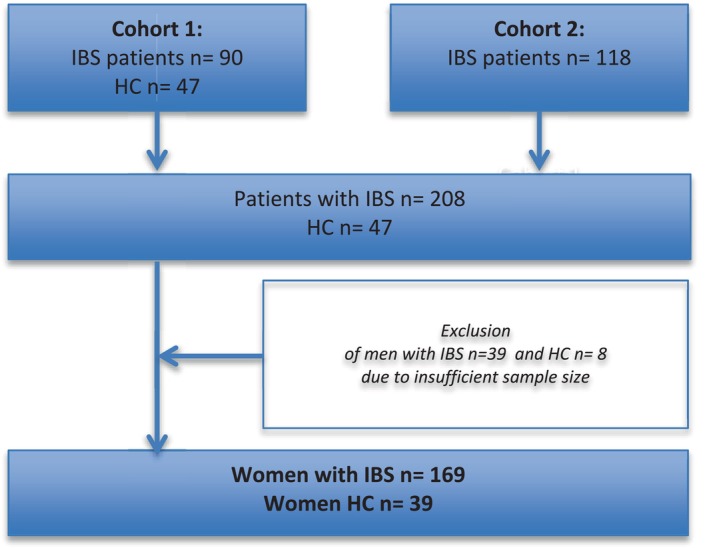
Study recruitment.

This study included data from two different study cohorts recruited via primary healthcare centers and referred to the gastroenterology clinic of Linköping University Hospital between 2014–2017 and 2021–2024 [21, 22]. The patients (18–65 years) with IBS fulfilled ROME III (the earlier cohort) and ROME IV (the later cohort) diagnostic criteria for IBS. Only patients with moderate or severe symptom burden measured with the IBS Severity Scoring System questionnaire (IBS‐SSS) were included. HCs without any history of gastrointestinal disease or complaints were recruited by local advertisement and offered monetary compensation. Participants were recruited consecutively. Due to the small numbers of recruited men with IBS and childhood trauma, only women were included in the final analysis. The participants in the control group had a similar age profile, although not matched 1:1 with the IBS group. The process of recruitment of the participants is presented in Figure 1. All procedures for this study were approved by the regional ethical research committee (Dnrs. 2013/506‐32; 2014/264‐32; 2019/029‐32).

### 2.2 Questionnaires

#### 2.2.1 CTQ

The concept of childhood trauma is commonly assessed with help of the Childhood Trauma Questionnaire (CTQ). CTQ is a 28‐item self‐administered tool used to identify and measure the prevalence of childhood trauma from a retrospective perspective. Twenty‐five questions address five types of maltreatments—physical, emotional, and sexual abuse, and physical and emotional neglect. The three remaining questions measure minimization/denial to determine false‐negative trauma reports. The answers are based on a 5‐point Likert scale and rated from “never true”(1) to “very often true” (5). Some of the questions are coded in reverse order [12, 13, 23]. The sum score indicates none or mild (≤ 35), moderate (36–50), and severe and extreme (> 51) childhood trauma. The cut‐off score for childhood trauma was 36. The cut‐off scores for having experienced different types of childhood trauma were the following: sexual abuse ≥ 5, physical abuse and physical neglect ≥ 7, emotional abuse ≥ 8, and emotional neglect ≥ 9 [13].

#### 2.2.2 GSD

Gastrointestinal Symptom Diary (GSD) was used by the women with IBS to record their gastrointestinal symptoms on 14 consecutive days [24, 25, 26]. The symptoms (abdominal pain, nausea, bloating) as well as every single bowel movement with stool consistency (defined by Bristol Stool Chart) and defecation symptoms (urgency and feeling of incomplete evacuation) were recorded. The mean frequency of symptom episodes per week (and proportions of defecation symptoms) and symptom duration (hours) per day were extracted from the diary data.

#### 2.2.3 IBS‐SSS

IBS Severity Scoring System (IBS‐SSS) evaluates overall IBS symptom severity by measuring five items: frequency and intensity of abdominal pain, severity of bowel distension/bloating, satisfaction with bowel habits, and interference with daily life [27]. Each item scores between 0 and 100, and the sum score indicates mild (75–175), moderate (175–300), or severe (> 300) symptoms.

#### 2.2.4 HADS

The Hospital Anxiety and Depression Scale (HADS) serves as a screening tool for current symptoms of anxiety and depression. HADS comprises two subscales, HADS‐A and HADS‐D, each containing 7 items. The score on each subscale ranges from 0 to 21 [28, 29]. Cut‐off values ≥ 8 indicate subclinical anxiety or depression, and values ≥ 11 are defined as indicating symptom severity of anxiety or depression within a clinically relevant range (caseness), respectively.

#### 2.2.5 PHQ‐15

Patient Health Questionnaire (PHQ‐15) was used to measure severity across 15 of the most common somatic symptoms (excluding upper respiratory tract symptoms) in outpatient settings [30]. The questionnaire is a useful tool for evaluating somatization. The severity of each individual symptom is scored between 0 (“not bothered at all”) and 2 (“bothered a lot”), and the total score ranges from 0 to 30.

#### 2.2.6 BPI

Brief Pain Inventory (BPI) is a pain assessment tool, which measures pain intensity (4 items) and pain interference in the patient's life (7 items) [31]. Each item is scored between 0 and 10. Pain severity scores range from 0 to 40 and pain interference from 0 to 70, with higher scores indicating higher severity and interference, respectively.

### 2.3 Statistical Analysis

Normality of distribution was controlled by Kolmogorov–Smirnov and by Shapiro–Wilk tests. The results are presented as mean and standard deviation for continuous data with normal distribution, as median and interquartile range, IQR, for continuous data with non‐normal distribution and frequency percentage for ordinal data. Significance of association was measured by Pearson Chi‐square for categorical variables; it was accepted at the level lower than 5% (*p* < 0.05). Assessment of the strength of association was measured with Phi or Cramer's V for nominal variables and with Gamma for ordinal variables. Mann–Whitney *U* test was used for group comparisons of non‐normally distributed interval data (*p* < 0.05). Additionally, bivariate and Spearman's correlation analyses were performed.

## 3 Results

### 3.1 Demographics

The total sample consisted of 208 women (39 HCs, 19%). The median age of women with IBS was 32 years (IQR = 47) and 31 years (IQR = 35) for HCs. The age difference between women with IBS and HCs was not statistically significant. The median of IBS‐SSS score was 347 (IQR = 101) in women with IBS.

### 3.2 Childhood Trauma and IBS

**FIGURE 2:**
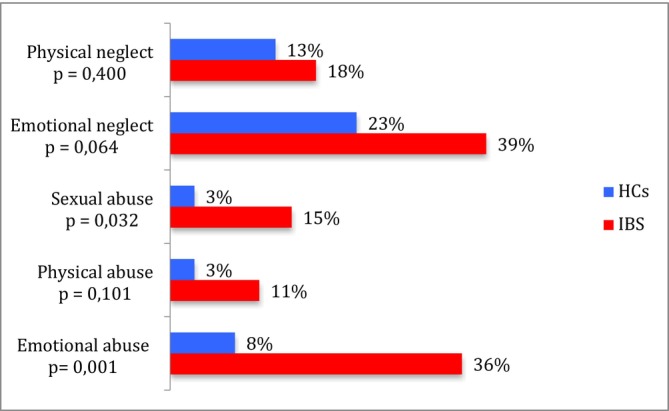
Prevalence of different types of childhood trauma in women with IBS and HCs.

The prevalence of both childhood trauma and severe childhood trauma was higher in women with IBS compared to HCs—38% vs. 15% (*p* = 0.007) and 14% vs. 0% respectively (*p* = 0.012). The prevalence of all five types of childhood trauma was higher in women with IBS compared to HCs. The most common types of childhood trauma in women with IBS were emotional neglect (39%) and emotional abuse (36%), while in HCs—emotional neglect (23%) and physical neglect (13%) were most common. There was a statistically significant difference in the prevalence of emotional abuse (36% and 8%, *p* = 0.001) and sexual abuse (15% and 3%, *p* = 0.032) in women with IBS and HCs (see Figure 2).

The bivariate analysis demonstrates that there is a moderate to strong association between all types of childhood trauma in women with IBS. The strongest association is seen between emotional abuse and emotional neglect (*r* = 0.717, *p* = 0.001) (Table 1).

**TABLE 1:** Bivariate correlations between different types of childhood trauma in women with IBS.

Spearman's rho	Emotional abuse	Physical abuse	Sexual abuse	Emotional neglect	Physical neglect
Emotional abuse	1.000	0.536**	0.389**	0.717**	0.594**
*p*	.	0.000	0.000	0.000	0.000
Physical abuse	0.536**	1.000	0.469**	0.393**	0.493**
*p*	0.000	.	0.000	0.000	0.000
Sexual abuse	0.389**	0.469**	1.000	0.304**	0.376**
*p*	0.000	0.000	.	0.000	0.000
Emotional neglect	0.717**	0.393**	0.304**	1.000	0.703**
*p*	0.000	0.000	0.000	.	0.000
Physical neglect	0.594**	0.493**	0.376**	0.703**	1.000
*p*	0.000	0.000	0.000	0.000	

*Note:* Significant * if *p* < 0.05 and ** if *p* < 0.01.

Experience of childhood trauma elevated the odds of having IBS (OR 3.41, 95% CI, 1.35–8.60, *p* = 0.009). All types of childhood trauma showed a trend of increasing odds of having IBS. However, only in the case of emotional abuse were the increased odds of IBS statistically significant—OR 6.69 (95% CI, 1.97–22.68, *p* = 0.002).

There was a weak positive correlation between CTQ total score and IBS‐SSS total score in women with IBS (*r* = 0.305, *p* = 0.016). IBS‐SSS total score positively correlated with emotional neglect in women with IBS (*r* = 0.262, *p* = 0.039); although the strength of the correlation was also weak.

Women with IBS with childhood trauma, compared to women with IBS without childhood trauma, reported a higher prevalence of defecation urgency—57% (IQR = 55) and 40% (IQR = 44) respectively (*p* = 0.027). No statistically significant differences were found between women with IBS with and without childhood trauma in terms of frequency and duration of nausea, abdominal pain, and bloating, nor as to the frequency of straining and incomplete evacuation (see Table 2).

**TABLE 2:** IBS‐related symptoms in case of childhood trauma and different types of childhood trauma in women with IBS.

	Nausea episodes/week, median	Nausea hours/week, median	Abdominal pain episodes/week, median	Abdominal pain, hours/week, median	Urgency/stool %, median	Straining/stool, median	Incomplete evacuation/stool, % median	Bloating episodes/week, median	Bloating hours/week, median
Childhood trauma	3.25	9.35	8	32.5	57	69	63	7	53
No childhood trauma	3.09	6.05	7.5	25.3	40	53	60	7	34
*p*	0.953	0.766	0.643	0.324	0.027*	0.105	0.998	0.117	0.517
Emotional abuse	3.5	7	9	37	56	69	66	7	62
No emotional abuse	3	8.5	7	23	40	53	60	7	46
*p*	0.298	0.423	0.228	0.029*	0.013*	0.052	0.094	0.485	0.653
Physical abuse	4.5	19	10	46.5	67	69	69	8	62
No physical abuse	3	7.5	7.5	28	41	54	61	7	46
*p*	0.074	0.150	0.205	0.283	0.004**	0.151	0.266	0.348	0.598
Sexual abuse	4	11	8	37	56	69	78	7	54
No sexual abuse	3	8	8	28	42	54	61	7	46
*p*	0.751	0.472	0.512	0.334	0.173	0.207	0.284	0.552	0.598
Emotional neglect	3	7	8	32	52	66	64	7	35
No emotional neglect	3	9	7.5	26	41	52	61	7	53
*p*	0.941	0.971	0.349	0.315	0.237	0.176	0.658	0.313	0.301
Physical neglect	4.5	11	9.5	31	57	64	73	8	50
No physical neglect	3	8	7.5	29	41	55	61	7	46
*p*	0.138	0.262	0.266	0.407	0.054	0.764	0.207	0.862	0.748

*Note:* Significant * if *p* < 0.05 and ** if *p* < 0.01.

The results indicate a stronger association between emotional abuse and specific gastrointestinal symptoms. Specifically, women with IBS and a history of emotional abuse reported a longer duration of abdominal pain compared to women with IBS without emotional abuse—37 (IQR = 53) and 23 (IQR = 43) hours per week respectively (*p* = 0.029). Women with IBS with emotional and physical abuse had a higher ratio of urgency per stool compared to women with IBS without these types of childhood trauma—56% (IQR = 55%) and 40% (IQR = 45%), *p* = 0.013 in the case of emotional abuse, and 67% (IQR = 47%) and 41% (IQR = 47%), *p* = 0.004 in the case of physical abuse. No statistically significant differences were found with respect to nausea, abdominal pain frequency, straining, incomplete evacuation, and bloating when comparing women with IBS with and without different types of childhood trauma (see Table 2).

### 3.3 Childhood Trauma, Psychological, and Extraintestinal Symptoms

Anxiety was more common in women with IBS with childhood trauma when compared to women with IBS without childhood trauma—60% and 41% respectively (*p* = 0.023). Anxiety was reported by 47% of women with IBS with a history of emotional abuse, compared to 27% of women with IBS without emotional abuse (*p* = 0.009). The prevalence of anxiety in women with IBS with emotional neglect, and in women with IBS without emotional neglect was 48% and 31% respectively (*p* = 0.023).

Depression was, on average, less commonly reported when compared to anxiety in all studied populations. There was a higher prevalence of depression in women with IBS and childhood trauma compared to women with IBS without childhood trauma—27% and 11%, respectively (*p* = 0.009). 28% of women with IBS with emotional abuse reported depression compared to 12% of women with IBS without emotional abuse (*p* = 0.012). 50% of women with IBS with physical abuse reported depression compared to 12% of women with IBS without physical abuse (*p* = 0.001).

Experience of childhood trauma was associated with a higher burden of extraintestinal symptoms. The average total score of somatic symptoms according to PHQ‐15 was 17 points (IQR = 5) in women with IBS and childhood trauma and 14 points (IQR = 6) in women with IBS without childhood trauma, respectively (*p* = 0.001). There was a positive association between the total score of somatic symptoms and childhood trauma total score (CTQ) (*r* = 0.249, *p* = 0.002). The average somatic symptoms total score was higher in women with IBS with all five types of childhood trauma compared to women with IBS without these types of childhood trauma. These differences were statistically significant in all five cases (see Table 3).

**TABLE 3:** Types of childhood trauma, somatic symptoms, pain intensity, and pain interference in women with IBS.

	Childhood trauma	Emotional abuse	Physical abuse	Sexual abuse	Emotional neglect	Physical neglect
*PHQ‐15*, total somatic symptoms score, mean						
Women with IBS with particular trauma type	17	17	19	19	17	18
Women with IBS without particular trauma type	14	15	15	15	15	15
*p*	0.0001***	0.006**	0.004**	0.0001***	0.021*	0.007**
*BPI pain intensity*, mean						
Women with IBS with particular trauma type	18	20	25	25	18	19
Women with IBS without particular trauma type	15	14	15	15	15	15
*p*	0.016*	0.008**	0.0001***	0.0001***	0.461	0.017*
*BPI pain interference*, mean						
Women with IBS with particular trauma type	37	40	51	42	40	43
Women with IBS without particular trauma type	27	27	29	29	27	28
*p*	0.003**	0.013*	0.0001***	0.004**	0.019*	0.0001***

*Note:* Significant * if *p* < 0.05, ** if *p* < 0.01, and *** if *p* < 0.001.

Abbreviations: BPI, Brief Pain Inventory; PHQ‐15, Patient Health Questionnaire‐15.

Women with IBS and childhood trauma had higher general pain intensity when compared to women with IBS without childhood trauma—18 (IQR = 13) and 15 (IQR = 12) points respectively (BPI pain intensity score, *p* = 0.016) and higher pain interference—37 (IQR = 32) and 27 (IQR = 28) points respectively (BPI interference score, *p* = 0.003). Both the total pain intensity score and pain interference score respectively showed positive associations with the total childhood trauma score in women with IBS (*p* = 0.031 and *p* = 0.002). Women with IBS who experienced emotional, physical, and sexual abuse as well as physical neglect had higher pain intensity compared to women with IBS without these types of childhood trauma (*p* = 0.008, *p* = 0.0001, and *p* = 0.0001 respectively). Pain interference was higher in women with IBS compared to women without IBS in the case of all categories of childhood trauma, and the differences were statistically significant (see Table 3).

## 4 Discussion

The main findings of this study were: (1) childhood trauma and severe childhood trauma were more common in women with IBS compared to HCs; childhood trauma increased the odds of having IBS and positively correlated with IBS symptom severity; (2) childhood trauma was associated with a higher prevalence of specific gastrointestinal symptoms such as defecation urgency and extraintestinal symptoms—anxiety, depression, higher burden of somatic symptoms, and higher general pain intensity and interference; (3) emotional abuse and sexual abuse were more common in women with IBS compared to HCs, and emotional abuse was the strongest predictor of IBS; (4) women with IBS and emotional abuse had longer abdominal pain duration and more urgency, as well as higher anxiety scores; women with IBS and physical abuse had a higher prevalence of urgency and higher depression scores; (5) no differences between types of childhood trauma in relation to somatization and pain interference were found. Each of these points will be addressed below.

### 4.1 Childhood Trauma Was Commonly Reported by Women With IBS and Seems to Raise the Odds of IBS

The high self‐reported prevalence of childhood trauma and severe childhood trauma in women with IBS compared to HCs confirms the results of previous studies and indicates an important role of childhood trauma in IBS pathophysiology [7, 20]. The severity of childhood trauma was positively correlated with the severity of IBS symptoms. Therefore, both the experience and severity of childhood trauma appear to raise the odds of having IBS and contribute to worse outcomes.

The association between childhood trauma and IBS is likely to be complex, multifactorial, and is often discussed within the larger brain–gut–microbiome‐exposome framework. Its components include dysregulation of the HPA axis and stress response [4, 32], activation of the fear‐response related pathways [5], particular pain pathways, structural alterations in core brain structures involved in emotional regulation, chronic heightened awareness and hyperarousal [8, 18], immune dysregulation, dysfunction of the mucosal barrier [4], and changes in gene expression [33]. Experiencing trauma in early life is a high‐risk factor for permanent dysregulation of the HPA axis that can alter stress response and pain perception in adulthood [34]. Videlock et al. found higher salivary cortisol in response to sigmoidoscopy and slower return of stimulated cortisol to basal levels in patients with IBS with childhood trauma, which were associated with higher IBS symptom intensity and lower quality of life [32]. Gupta et al. argue that stress related to ACEs contributes to neuroplastic changes and modulates regional brain structures associated with mood and affect regulation [33]. Altered cortico‐limbic pain modulatory pathways in the case of childhood abuse contribute to linking hypervigilance and emotions such as fear and anxiety with visceral pain [35]. Animal studies indicate that early‐life stress in neonatal rodents has implications for gut dysfunction in adulthood by contributing to low‐grade inflammation, epithelial abnormalities, microbial translocation, and altered gastrointestinal microbiota [36, 37]. At the same time, social and psychological factors, such as confiding in others and active coping, may serve as protective mechanisms decreasing the odds of IBS and its severity [9].

### 4.2 Urgency to Defecation, Anxiety, Depression, Somatization, and General Pain Were Associated With Childhood Trauma

Prevalence and duration of all gastrointestinal symptoms—nausea, abdominal pain, urgency, straining, incomplete evacuation, and bloating—was higher in women with IBS with childhood trauma when compared to women with IBS without childhood trauma. However, only in the case of urgency was this difference statistically significant. Urgency is a multifaceted symptom related to several factors, such as stool consistency, visceral sensitivity, or stress [38]. In the present study, the definition of urgency was a sudden need to rush to the bathroom to empty bowels, as recorded prospectively in the 2‐week gastrointestinal diary. Since we do not have access to sensitivity measurements in all patients included in this study, we cannot rule out to what extent rectal hypersensitivity, stool consistency, or psychological factors mediated the significant associations between urgency and childhood trauma. Visceral sensitivity in IBS patients has been associated with altered activity in regions of the emotional arousal and default mode networks, regions that are also altered by childhood trauma regardless of diagnosis [39, 40]. This may indicate a central mechanism of neuromodulation in IBS women with childhood trauma that is partially responsible for the hypersensitivity and the feeling of urgency in these patients. In contrast to other research, the differences in abdominal pain in the case of childhood trauma cannot be completely confirmed. This can be related to the study design and the use of other tools to evaluate abdominal pain. In particular, the duration and frequency of abdominal pain, and not abdominal pain intensity as in other studies, was evaluated in this research [4, 9, 20].

Women with IBS and childhood trauma had a higher prevalence of extraintestinal symptoms, such as somatization, higher general pain intensity, anxiety, and depression. A strong association between somatization and visceral hypersensitivity in IBS has been discussed by Grinsvall et al. [41]. Experience of adverse early‐life experiences may intensify this relationship. Schubach et al. argue that somatization mediates the relationship between childhood abuse and IBS symptoms, in particular abdominal pain and bloating [42]. This is explained by a potentially higher vigilance to somatic cues and altered modulation of mechanisms controlling visceral sensations in persons who experienced childhood trauma [42]. Our findings indicate that women with IBS and childhood trauma experience not only more severe IBS symptoms but also reduced mental wellbeing.

### 4.3 Emotional and Sexual Abuse Were Common in Women With IBS. Emotional Abuse Was a Strong Predictor of IBS

The prevalence of all types of childhood trauma was higher in women with IBS compared to HCs. However, only in the case of emotional and sexual abuse was this difference significant. This finding is consistent with the results of previous studies [7, 18, 20].

Childhood emotional abuse is defined as adverse parental behavior which produces damage to a child's emotional and psychological functioning [43]. It involves verbal behavior aimed to humiliate, degrade, or frighten. Emotional abuse was the only type of childhood trauma that increased the odds of having IBS. The odds of having IBS were almost six times higher in the case of emotional abuse. The strong association between emotional abuse and IBS is confirmed in a systematic review by Zia et al. [44].

Sexual abuse has been discussed as another important type of childhood abuse that increases odds of IBS in female patients [20]. This study cannot fully confirm it; however, it confirms a higher prevalence of sexual abuse in women with IBS. It can be speculated that sexual abuse is a more severe form of childhood trauma, which potentially intensifies IBS symptoms either directly or indirectly through increased psychological distress.

### 4.4 Emotional Abuse Was Associated With Longer Abdominal Pain Duration, Urgency, Anxiety, and Depression. Physical Abuse Is Associated With Higher Urgency and Depression

Women with IBS and emotional abuse experience longer abdominal pain duration and urgency as well as higher anxiety compared to women with IBS without emotional abuse. The experience of emotional abuse may have a stronger effect on the brain structures responsible for emotional regulation. As discussed earlier, modulation of brain regions related to emotional regulation has been associated with IBS [33, 45]. Rahal et al. suggest that early adverse life events can lead to impairments to detect, process, and modulate sensory information and generate appropriate autonomic behavioral responses [45].

Emotional abuse can also be an independent risk factor for IBS development related to poorer coping strategies. A person who has been ridiculed or humiliated is less likely to confide in others, which is a protective factor in the development of IBS [45].

The experience of physical abuse was related to a higher prevalence of urgency and depression in women with IBS. A history of physical abuse has been linked to more severe pain, higher severity of extraintestinal symptoms, and psychological comorbidities in IBS [41, 46].

### 4.5 No Differences as to Somatization and Pain Interference in Case of Different Types of Childhood Trauma Were Found

No differences between different types of childhood trauma in relation to somatic symptoms and pain interference were found in women with IBS with and without specific categories of childhood trauma. All types of childhood trauma contribute to a higher burden of somatic symptoms and pain interference, which may indicate that the general experience of distress or abuse in childhood is related to higher levels of somatization and higher visceral and central sensitivity. A higher somatization level, regardless of childhood trauma type, is discussed by Dutcher et al., who demonstrate that a higher level of each type of childhood maltreatment correlates with higher levels of somatic symptoms [47]. Women with IBS with certain types of childhood trauma—emotional, physical, and sexual abuse—have higher pain intensity. A potential explanation is that abuse, in contrast to neglect, is always intentional and implies a higher level of aggression toward another person; therefore, it potentially leads to a more traumatizing experience. Our finding can indicate a relationship between higher pain intensity and trauma severity, which has been discussed earlier.

To summarize, our study confirms that childhood trauma and severe childhood trauma are much more prevalent in women with IBS compared to HCs. Emotional neglect and emotional abuse were the most common types of childhood trauma in women with IBS, while HCs more often reported emotional and physical neglect. While some specific associations between sexual and physical abuse with gastrointestinal and extraintestinal symptoms were observed, the findings of this study highlight a stronger link between IBS and emotional abuse. Emotional abuse was the only type of childhood trauma that considerably increased the odds of IBS. These findings suggest that emotional abuse may play a significant role in the pathophysiology of IBS, which warrants further investigation. Black et al. suggest that a more complex subtyping of IBS patients based on bowel habits, extraintestinal symptoms, and psychological comorbidities can be beneficial for a more personalized and efficient treatment [48]. In this respect, childhood trauma and its subgroups can be considered as a criterion for such classification.

## 5 Strengths and Limitations

This study not only confirms the findings of previous research on the association between adverse childhood experiences and IBS, but also is one of the few to explore the differences between different types of childhood trauma. The strength of this study is that it examines both symptoms directly related to IBS and those associated with IBS—anxiety and depression, general pain, and somatization. The study used gastrointestinal symptom diaries to minimize recall bias in terms of IBS key symptoms. This study is the first of its kind in the Scandinavian context.

Some of the limitations of this study are recall bias as to the retrospective assessment of childhood trauma and the potential risk of over‐ or underreporting of traumatic experiences. A potential limitation is the limited definition of childhood trauma, which mostly focuses on child maltreatment and does not take into consideration other types of stressful early‐life experiences. The cross‐sectional design of the study, by nature, does not allow the analysis of causal relations and specific pathophysiological pathways. Due to challenges in the recruitment of a sufficient number of male participants, sex differences in relation to childhood trauma and IBS were not studied. The comparison groups were unequal in terms of size. Diet, genetics, lifestyle, and other potential confounding variables were not included in the analysis. The study population includes patients with medium and high IBS burden, which is not representative of the broader population of IBS patients. Due to the explorative nature of this study, more detailed interconnections between IBS and different types of childhood trauma as well as IBS and heaviness trauma burden (i.e., exposing to several childhood trauma types) were now assessed, which opens up potential for further research. The parenting styles and legal implications of child abuse reflect Scandinavian context and vary across the world. The disclosing of experiences of childhood trauma, along with resilience and coping strategies, are important factors that can contribute to a better understanding of the association between childhood trauma and IBS, but which were not examined in the current study.

## 6 Conclusion

The pathophysiology of IBS is complex and multifactorial. Traumatic early‐life experiences are a component of the broader exposome that increases the risk of developing IBS and exacerbates its outcomes. The association between specific types of childhood trauma and IBS varies. This study highlights a particularly stronger association between IBS—along with symptoms such as prolonged abdominal pain episodes and urgency, as well as IBS‐associated comorbidities—and emotional abuse. Further research is necessary to deepen the understanding of this relationship and to potentially identify a distinct IBS phenotype in individuals with a history of childhood trauma and emotional abuse. Given the high prevalence of childhood trauma among IBS patients, an important practical implication of these findings is the need for routine screening for childhood trauma and the provision of appropriate psychopharmacological interventions by healthcare providers to enhance the effectiveness of IBS treatment.

## Author Contributions

Conceptualization: S.W., A.I., and T.B. Formal analysis: T.B. Investigation and methodology: S.W., J.S., O.Be., N.B., and T.B. Project administration, supervision: S.W. Writing – original draft: T.B. Writing – review and editing: T.B., S.W., A.I., J.S., G.O., O.Be., N.B., R.S., and O.Bo.

## Conflicts of Interest

The authors declare no conflicts of interest.

## Data Availability

The data that support the findings of this study are available on request from the corresponding author. The data are not publicly available due to privacy or ethical restrictions.
